# The association between vitamin D receptor polymorphism and phases of chronic hepatitis B infection in HBV carriers in Thailand

**DOI:** 10.1371/journal.pone.0277907

**Published:** 2022-12-09

**Authors:** Prooksa Ananchuensook, Sirinporn Suksawatamnauy, Panarat Thaimai, Supachaya Sriphoosanaphan, Kessarin Thanapirom, Chinachote Teerapakpinyo, Yong Pooworawan, Piyawat Komolmit

**Affiliations:** 1 Division of Gastroenterology, Department of Medicine, Faculty of Medicine, Chulalongkorn University, Bangkok, Thailand; 2 Center of Excellence in Liver Diseases, King Chulalongkorn Memorial Hospital, Thai Red Cross Society, Bangkok, Thailand; 3 Research Unit of Hepatic Fibrosis and Cirrhosis, Department of Medicine, Chulalongkorn University, Bangkok, Thailand; 4 Chula GenePRO Center, Research Affairs, Faculty of Medicine, Chulalongkorn University and The King Chulalongkorn Memorial Hospital, Bangkok, Thailand; 5 Center of Excellence in Clinical Virology, Faculty of Medicine, Chulalongkorn University, Bangkok, Thailand; Kaohsiung Medical University Hospital, TAIWAN

## Abstract

Vitamin D receptor (VDR) polymorphism partly regulates the immune system and is associated with hepatic flare in chronic Hepatitis B virus infection (HBV). Our study identified the association between two distinct phases, VDR polymorphisms and HBV inactive carrier (IC) and chronic hepatitis (CH). Chronic HBV patients were enrolled from February to August 2020. An HBV viral load (VL) < 2,000 IU/ml twice for 6 months apart, with no prior history of HBV treatment, defined the IC phase. Six common polymorphisms in the *VDR* gene, including *CdX-2*, *GATA*, *Fok*I, *Bsm*l, *Apa*I, and *Taq*I, were studied using real-time PCR. The different outcomes in allele, genotype, and haplotype frequencies in between groups and linkage disequilibrium (LD) mapping were analyzed. Among 324 enrolled patients, there were 163 patients in IC and 161 patients in CH phases. The mean vitamin D levels were not statistically different between groups. The proportion of allele frequencies of *CdX-2* in IC and CH was 53.7% and 62.7% for G allele, and 46.3% and 37.3% for A allele (p 0.019). The proportion of GG genotype of *CdX-2* was less frequently found in patients with IC compared to that in patients with CH (27% vs 41%, p 0.028). By multivariate analysis, *CdX-2* G/A genotypes were independently associated with IC, with adjusted odd ratio (OR) 1.83 (1.10–3.04), p 0.019. The LD mapping of single nucleotide polymorphisms (SNP) revealed high LD scores in *Bsm*l/*Apa*I/*Taq*I (BAT) haplotype in both groups while, *CdX-2/GATA* and *GATA/Fok*I demonstrated high LD score only in CH group. *CdX-2* G/A genotypes were independently associated with IC status in Thai patients with chronic HBV infection. The difference in LD of the *CdX-2/GATA* and *GATA*/*Fok*I haplotypes in between groups may represent a non-random selection resulting in the variation of immune control.

## Introduction

In 2015, WHO reported 257 million people (3.5%) with hepatitis B (HBV) infection globally [1]. In addition, the estimated prevalence of HBV infection was 3.9%, or 291 million patients, according to the 2016 Polaris Observatory study [2]. The natural history and risk of liver-related complications, such as cirrhosis and hepatocellular carcinoma (HCC), among HBV-infected patients are associated with differences in immune control. Based on the prognosis, phases of HBV infection may be categorized into inactive carrier (IC) and chronic hepatitis (CH). IC is defined by a low level of HBV DNA, the absence of Hepatitis B “e” antigen (HBeAg), and a lack of evidence for hepatic inflammation and fibrosis. While CH is defined by a persistent HBV viral load (VL) > 2,000 IU/ml with evidence of hepatic inflammation or fibrosis requiring antiviral therapy. Chen et al. demonstrated that risks of HCC and cirrhosis depend on HBV DNA level regardless of HBeAg status [3, 4]. Therefore, IC is associated with better prognosis than CH [5, 6].

Apart from inducing calcium homeostasis, vitamin D also regulates the host immune status via the vitamin D receptor (VDR). Vitamin D binds to VDR, generating a heterodimer with a retinoid X receptor (RXR), that facilitates the transcription of proteins functioning in the immune system [7, 8]. *VDR* is located in the long arm of chromosome 12. Although many single nucleotide polymorphisms (SNPs) of *VDR* have been reported, the common SNPs reported are *CdX-2*, *GATA*, *Fok*I, *Bsm*l, *Apa*I, and *Taq*I [9, 10]. Previous studies have reported an association between *VDR* polymorphisms and severe hepatitis flare-ups, decompensated cirrhosis, and hepatoma in patients with chronic HBV infection [11–13]. However, the relationship between *VDR* polymorphisms and IC status remains unclear.

The objective of our study was to assess the influence of VDR polymorphism on the two HBV infection phases, IC and CH, in Thai patients with chronic HBV infection.

## Materials and methods

### Participants

Thai patients with chronic HBV infection, aged 30–60 years, who presented at an outpatient liver clinic of King Chulalongkorn Memorial Hospital (KCMH) from February to October 2020 were enrolled in this study. Chronic HBV infection of at least 6 months was confirmed by HBsAg seropositivity. The IC phase was defined by an HBV viral load of less than 2,000 IU/ml in two samples collected 6 months apart, without a history of HBV treatment. The CH phase was defined by an HBV viral load (VL) > 2,000 IU/ml with evidence of hepatic inflammation or fibrosis requiring HBV treatment, according to AASLD guidelines [6]. Hepatic inflammation was defined by an alanine aminotransferase (ALT) level > 40 IU/ml. Exclusion criteria included pregnancy, co-infection with hepatitis C virus or human immunodeficiency virus (HIV), decompensated liver cirrhosis, comorbidities requiring immunosuppressive drugs, and active infections such as tuberculosis, hepatocellular carcinoma, and other cancers.

### Data collection

Demographic data, including age, sex, height, and weight, were recorded. The HBV viral load, ALT, HBeAg, and evidence of liver fibrosis were obtained from the KCMH database within 1 year of blood collection. Liver cirrhosis and fibrosis were assessed via radiological examination or transient elastography (FibroScan 502, France). The study protocol was approved by the Institutional Review Board of the Faculty of Medicine of Chulalongkorn University (number 745/62). Informed consent was obtained from all participants. Blood samples of 12 ml was collected from each participant, in ethylenediaminetetraacetic acid (EDTA) tubes and stored at 4°C until vitamin D levels and VDR genotypes were analyzed. Our study was registered with the Thai Clinical Research Registry (TCTR20191110003).

### Vitamin D assays

The 25(OH) vitamin D levels in plasma samples were measured using a Liaison 25 (OH) vitamin D total assay (DiaSorin, Saluggia, Italy), which was performed using a LIAISON^®^ chemiluminescence analyzer according to the manufacturer’s instructions. The final concentration was reported in ng/mL.

### Genotype analysis

Blood samples were collected in EDTA tubes at 4°C. Genomic DNA was extracted from a 200 μl buffy coat of the blood samples via an Exgene kit (Cat No.106-101, Gene All, Korea) following the manufacturer’s instructions. Next, 20 μl of proteinase K was added to the samples and mixed with 200 μl of buffer BL and 200 μl of absolute ethanol. Then, 600 μl of buffer BW, 700 μl of buffer TW, and 200 μl of buffer AE were added in order. Pure genomic DNA was obtained following centrifugation and incubation.

Five vitamin D receptor polymorphisms, *Cdx-2*, *GATA*, *Fok*I, *Apa*I, and *Taq*I, were identified via a real-time polymerase chain reaction (RT-PCR) using a commercial TaqMan genotyping assay with minor groove binder (MGB) probes (Cat No.4351379; Applied Biosystems, NY, USA). The real-time PCR mixture consisted of 1 μl of DNA extract, 0.5 μl of 20x TaqMan SNP genotyping assay (Applied Biosystems), and 5 μl of 2x TaqMan genotyping master mix (Applied Biosystems) adjusted to a final volume of 10 μl with distilled water. The PCR conditions were as follows: 10 min at 95°C; 40 cycles of 15 s each at 95°C; and 60 s at 60°C. A fluorescent signal was detected at the end of each cycle. The genotypes of rs11568820 (cdx-2), rs4516035 (GATA), rs2228570 (FokI), rs7975232 (ApaI), and rs731236 (TaqI) were analyzed using QuantStudio^™^ software (ViiA^™^7) (Applied Biosystems, CA, USA).

The rs1544410 (*Bsm*I) polymorphism of vitamin D receptors was identified via DNA sequencing. Further, 4 μl of DNA samples were used to set up 25 μL PCRs using the AccuStart^™^ II GelTrack PCR SuperMix (Quanta Biosciences, Gaithersburg, MD, USA) and the primer sets, F: 5’-CTC ACT GCC CTT AGC TCT GC-3’ and R: 5’-TTG GAC CTC ATC ACC GAC AT-3’. The final concentration of each primer was 0.2 μM.

The amplification reaction was carried out under the following conditions: pre-incubation at 94°C for 3 min; followed by 35 cycles comprising denaturation at 94°C for 30 s; primer annealing at 58°C for 30 s; extension at 72°C for 30 s; and a final extension at 72°C for 7 min. The expected 357 base pair PCR products were analyzed by gel electrophoresis. The 2% Agarose gel was stained with MaestroSafe^™^ (LABGENE Scientific, Switzerland) and visualized under UV light.

#### Direct DNA sequencing

The purified DNA and same primer used for PCR amplification served as templates for DNA sequencing performed by First BASE Laboratories Sdn Bhd (Selangor Darul Ehsan, Malaysia). The nucleotide sequences were analyzed in both directions using forward and reverse primers to confirm. The sequences were analyzed using CHROMAS LITE v.2.0 (www.technelysium.com.au).

#### BsmI polymorphism analysis

The nucleotides were compared with reference sequences of *VDR*, rs1544410, available at the NCBI (National Center for Biotechnology Information) GenBank database using the BLAST (Basic Local Alignment Search Tool) program (http://blast.ncbi.nlm.nih.gov/Blast.cgi).

### Control population for IRB

The control population consisted of volunteers among blood donors at multiple sites of the National Blood Center, Thai Red Cross Society (n = 99), and HBsAg negative visitors who presented for health check-ups at King Chulalongkorn Memorial Hospital (n = 84). All subjects were of Thai ethnic origin by self-admission.

### Ethical statement

The study was approved by the Institutional Review Board of the Faculty of Medicine, Chulalongkorn University, Bangkok, Thailand (IRB No. 431/58). Written informed consent was obtained from all participants. All methods were performed in accordance with the relevant guidelines and regulations.

### Statistical methods

#### Epidemiologic data

Statistical analyses were performed using SPSS software (version 22.0; IBM Corp., Armonk, NY, USA). Categorical variables are presented as frequencies (%) while continuous variables are expressed as the mean and standard deviation. Comparison of cases and controls was performed using the chi-square test, Fisher’s exact test, and independent sample t-tests for categorical and continuous variables, respectively.

The Hardy-Weinberg equilibrium (HWE) of genotype frequency among participants, linkage disequilibrium (LD), haplotype construction, and genetic association were analyzed using SHEsis Online Version (http://analysis.bio-x.cn/myAnalysis.php) [14, 15]. P-values for allele, genotype and haplotype frequencies were analyzed using Chi-square, while the p-value for HWE (pHWE) was calculated using Fisher’s exact test. Significant pHWE meant a departure from HWE [16]. Linkage disequilibrium (LD) showed the non-random association between alleles at two loci [17]. The degree of LD between SNPs is represented by D’ and considered well above 0.8 [18].

#### Sample size calculation

The sample size required for an unmatched case–control study was calculated using the n4studies program. Based on a study conducted on vitamin D receptor polymorphism and IC, we applied the prevalence of ApaI SNPs in patients with hepatitis flare (40.9%) [13]. We intended to enroll 161 patients with IC and 161 patients with CH to achieve 80% power when detecting the minimum odds ratio (OR) at 0.5% significance with a default rate of 5%. The Chi-square test was performed to evaluate the HWE of the studied genetic polymorphisms compared to 300 healthy controls.

## Results

### Patient characteristics

A total of 324 HBV-infected patients, including 163 with IC and 161 with CH, were enrolled in our cohort during the study period. The mean age of the patients with IC and CH was 48.5 ± 7.9 and 48.6 ± 8.3 years, respectively (p = 0.953). The proportion of male patients was significantly lower in the IC group. Among patients with CH, 40.5% and 13.0% were seropositive for HBeAg and cirrhosis, respectively. No statistical difference was observed between the mean vitamin D levels of the IC and CH groups. The proportions of patients with vitamin D insufficiency and deficiency in the IC and CH groups were 75.3% and 82.0%, respectively. Baseline characteristics of the participants are presented in Table 1.

**Table 1:** Baseline characteristics of patients with HBV inactive carrier (IC) and chronic hepatitis (CH) phases.

Characteristics	IC, N = 163	CH, N = 161	p-value
	N (%), Mean ± SD	N (%), Mean ± SD	
Mean age (years)	48.57 ± 7.99	48.62 ± 8.33	0.953
Sex: Male	75 (46.0%)	110 (68.3%)	< 0.001*
Vitamin D level (ng/mL)	23.41 ± 12.08	22.76 ± 10.18	0.609
Vitamin D status			
• Normal (≥ 30 ng/mL)	37 (24.7%)	28 (18.0%)	0.164
• Insufficiency (20–29.99 ng/mL)	35 (23.3%)	54 (34.6%)	0.033
• Deficiency (< 20 ng/mL)	78 (52.0%)	74 (47.4%)	0.493
HBV DNA (IU/mL)	530.3 ± 583.46	45920162.4 ± 13995169.2	< 0.001*
log	2.72 ± 2.76	7.66 ± 7.15	
HBeAg			< 0.001*
• Positive	0 (0%)	62/153 (40.5%)	
• Negative	163/163 (100%)	91/153 (59.5%)	
Cirrhosis	0	21/161 (13.0%)	

*Significant p-value ≤ 0.05.

### Allele frequencies of VDR SNPs

Allele frequencies of six VDR SNPs, *CdX-2*, *GATA*, *Fok*I, *Bsm*l, *Apa*I, and *Taq*I, are shown in Table 2. The proportion of allele frequency of *CdX-2* in IC and CH was 53.7% and 62.7% for the G allele and 46.3% and 37.3% for the A allele, which was statistically significant (p = 0.019).

**Table 2:** Allele and genotype frequencies of VDR SNPs in HBV inactive carrier and chronic hepatitis phases.

SNPs	Allele	Frequency (%)		p-value	OR (95%CI)	Genotype	Frequency (%)		p-value	p^HWE^
		IC^a^	CH^b^				IC	CH		
*CdX-2*	G	53.7	62.7	0.019*	0.68 (0.49–0.93)	G/G	27.0	41.0	0.028*	0.349
	A	46.3	37.3			G/A	53.4	43.5		
						A/A	19.6	15.5		
*GATA*	G	4.3	3.7	0.712	1.12 (0.51–2.48)	G/A	8.6	7.5	0.706	0.566
	A	95.7	96.3			A/A	91.4	92.5		
*Fok*I,	T	44.8	46.0	0.763	0.95 (0.70–1.30)	T/T	19.0	19.3	0.911	0.591
	C	55.2	54.0			T/C	51.5	53.4		
						C/C	29.4	27.3		
*Bsm*l	G	92.3	92.9	0.798	0.92 (0.51–1.66)	G/G	85.1	86.3	0.544	0.306
	A	7.7	7.1			G/A	14.9	13.0		
						A/A	0	0.6		
*Apa*I	T	77.9	74.5	0.312	1.20 (0.83–1.72)	T/T	57.1	51.6	0.478	0.006
	G	22.1	25.5			T/G	41.7	46.0		
						G/G	1.2	2.5		
*Taq*I	T	92.9	94.4	0.443	0.80 (0.42–1.51)	T/T	85.9	89.4	0.316	0.332
	C	7.1	5.6			T/C	14.1	9.9		
						C/C	0	0.6		

^a^inactive carrier;

^b^chronic hepatitis

*Significant p-value ≤ 0.05

### Genotype frequencies of VDR SNPs

The proportion of the GG genotype of *CdX-2* found in patients with IC was less than that in patients with CH (27% vs. 41%; p = 0.028). However, the mean vitamin D levels in patients with the GG, GA, and AA genotypes of *CdX-2* were not statistically different (Table 3). The highest proportions of AA, TC, GG, TT, and TT genotypes for *GATA*, *Fok*I, *Bsm*l, *Apa*I, and *Taq*I in both IC and CH, are summarized in Table 2.

**Table 3:** Vitamin D levels according to *CdX-2* genotypes in HBV inactive carrier and chronic hepatitis phases.

SNPs	Genotype (n IC^a^, CH^b^)	Vitamin D level (mean ± SD)		p-value
		IC	CH	
*CdX-2*	G/G (38, 64)	24.00 ± 12.75	21.79 ± 8.98	0.310
	G/A (83, 68)	23.04 ± 12.38	23.99 ± 11.97	0.638
	A/A (29, 24)	23.71 ± 10.58	21.87 ± 7.22	0.474

^a^inactive carrier;

^b^chronic hepatitis

*Significant p-value is ≤ 0.05

### Haplotype frequencies of VDR SNPs

**Fig 1:**
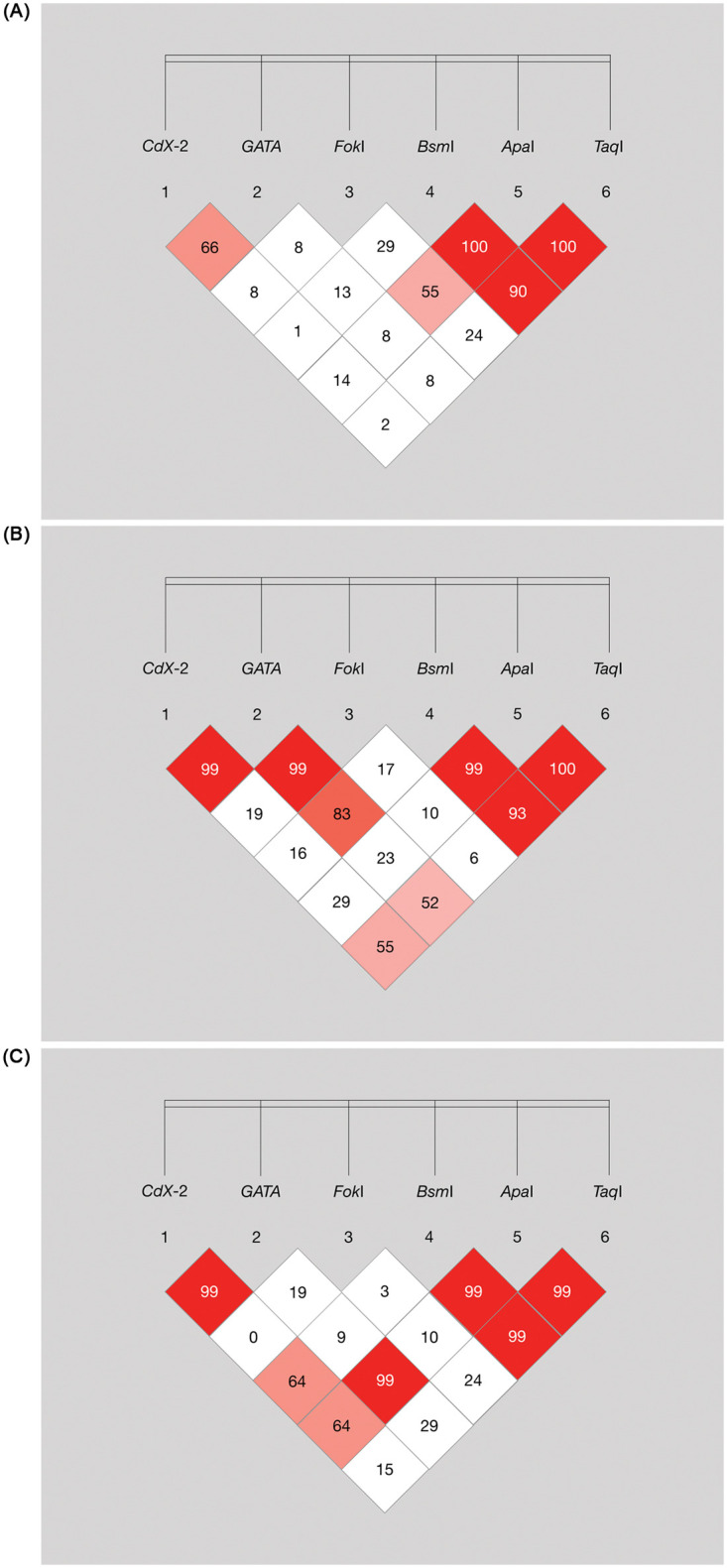
Genomic organization and linkage disequilibrium (LD) mapping of six SNPs of vitamin D receptor (*VDR*) gene. Patients with HBV inactive carrier (IC), chronic hepatitis (CH) phases and the general population.

*Bsm*I, *Apa*I, and *Taq*I showed high LD scores (D’ > 0.8) in both IC and CH groups. However, *CdX-2/GATA* and *GATA/FokI* demonstrated high LD scores only in the CH group (Fig 1). The haplotype frequencies of *CdX-2/GATA*, *CdX-2/GATA/Fok*I, *and Bsm*I*/Apa*I*/Taq*I are shown in Table 4. AA haplotype (*CdX-2/GATA)* and AAC haplotype (*CdX-2/GATA/FokI*) were significantly associated with an odds ratio (OR) of 1.43 (95%CI (1.04–1.96); p = 0.025) and an OR of 1.98 (95%CI (1.34–2.91); p < 0.001), respectively. However, the frequency of the GGC haplotype (*CdX-2/GATA/Fok*I) in IC was significantly lower than that in CH (OR = 0.12; 95%CI (0.02–0.69); p = 0.004). *Bsm*I*/*ApaI*/Taq*I haplotype frequencies indicated that GGT was the most frequently found haplotype in both groups. However, there was no significant difference between the haplotypes in IC and CH.

**Table 4:** Haplotype frequencies of VDR SNPs in HBV inactive carrier and chronic hepatitis phases.

Haplotypes	Frequency (%)		p-value	OR	95%CI
	IC^a^	CH^b^			
*CdX-2/GATA*					
GG	3.6	3.7	0.958	0.97	0.43–2.21
GA	50.1	59.0	0.027	0.70	0.51–0.96
AG	0.7	0	-	-	-
AA	45.6	37.3	0.025*	1.43	1.04–1.96
*Corrected significant p-value is ≤ 0.0250.					
*GATA/FokI*					
GT	2.1	0	-	-	-
GA	2.2	3.7	0.261	0.58	0.22–1.50
AT	42.7	46.0	0.545	0.90	0.66–1.24
AC	53.1	50.3	0.325	1.16	0.85–1.59
Corrected significant p-value is ≤ 0.0250.					
*CdX-2/GATA/FokI*					
GGT	2.7	0	-	-	-
GGC	0.5	3.7	0.004**	0.12	0.02–0.69
GAT	23.3	24.8	0.878	0.97	0.67–1.39
GAC	27.2	34.3	0.103	0.75	0.54–1.05
AAT	18.8	21.2	0.597	0.90	0.61–1.32
AAC	26.4	16.1	<0.001**	1.98	1.34–2.91
AGC	1.1	0	-	-	-
**Corrected significant p-value is ≤ 0.0167.					
*BsmI/ApaI*					
GT	70.2	67.4	0.432	1.14	0.81–1.59
GG	22.1	25.5	0.312	0.83	0.57–1.19
AT	7.7	7.1	0.798	1.08	0.59–1.94
Corrected significant p-value is ≤ 0.0250.					
*ApaI/TaqI*					
TT	70.9	68.9	0.595	1.09	0.78–1.53
TC	7.1	5.6	0.443	1.28	0.67–2.42
GT	22.1	25.5	0.312	0.83	0.57–1.19
Corrected significant p-value is ≤ 0.0250.					
*BsmI/ApaI/TaqI*					
GTT	69.6	67.1	0.516	1.11	0.79–1.57
GTC	0.6	0.3	-	-	-
GGT	22.1	25.5	0.299	0.82	0.57–1.18
ATT	1.2	1.9	-	-	-
ATC	6.4	5.3	0.536	1.23	0.63–2.38
AGT	0	0.1	-	-	-
Corrected significant p-value is ≤ 0.0167.					

^a^inactive carrier;

^b^chronic hepatitis

*Corrected significant p-value is ≤ 0.0250.

**Corrected significant p-value is ≤ 0.0167.

### Predictors of the IC phase

The results of univariate and multivariate analyses of the HBV IC phase are shown in Table 5. Sex and *CdX-2* genotypes were significantly associated with IC. Factors ≤ 0.05, were included in the multivariate analysis. The multivariate analysis indicated that the male gender and *CdX-2* G/A genotypes were independently associated with IC, with adjusted ORs of 0.40 (95%CI (0.25–0.63); p < 0.001) and 1.83 (95%CI (1.10–3.04); p = 0.019), respectively.

**Table 5:** Univariate and multivariate regression analysis of factors associated with HBV inactive carrier (IC) phase.

	Univariate analysis		Multivariate analysis	
	OR (95%CI)	p-value	OR (95%CI)	p-value
Age	0.99 (0.96–1.02)	0.806		
Sex (male)	0.39 (0.25–0.62)	< 0.001*	0.40 (0.25–0.63)	< 0.001*
*CdX-2* genotype				
• G/G	-	0.029*	-	0.045*
• G/A	1.84 (1.12–3.02)	0.016*	1.83 (1.10–3.04)	0.019*
• A/A	1.98 (1.03–3.77)	0.038*	1.85 (0.95–3.58)	0.068
Vitamin D level	1.00 (0.98–1.02)	0.608		

*Significant p-value ≤ 0.05

## Discussion

To the best of our knowledge, this is the first study that demonstrated the association between VDR polymorphism and the two distinct chronic HBV infection phases, IC and CH. In our case–control study, there were significant differences between the proportions of the allele and genotype frequencies of *CdX*-2 in IC and CH. In addition. the G/A genotype of *CdX*-2 showed significant association with the IC phase in the Thai patient with chronic HBV infection.

*CdX-2*, located in the 5’ region of *VDR*, acts as a modulator of promoter activity and influences *VDR* transcription. Arai et al. reported that the *CdX-2-*G allele showed 30% less transcriptional activity than the *CdX-2-A* allele, resulting in different bone mineral densities among postmenopausal women in Japan [19]. Apart from osteoporosis, *CdX-2* polymorphism also plays an important role in immune-related conditions. *CdX-2* AA is associated with an increased risk for tuberculosis and overall risk for cancer [20, 21]. Modulation of transcription activity, as described in previous studies, may explain the findings of our study that revealed the *CdX-2* G/A genotype as a potential predictor of IC status. In our study, there were no statistically significant differences between the vitamin D levels corresponding to *CdX-2* genotypes in the IC and CH groups. It is possible that *the CdX-2* G/A genotype may regulate *VDR* expression, regardless of vitamin D levels, which requires further study.

The AA-haplotype of *CdX-2*, *GATA*, and AAC-haplotype of *CdX-2*, *GATA*, and *Fok*I showed statistically significant associations with IC. Together with haplotype, triangular LD mapping suggested that there was a resemblance between the mapping status of IC haplotypes *CdX-2/GATA* and *GATA/Fok*I and those of the general population. Both *CdX*-2 and *GATA* are located in the promotor region [22]. Previous evidence also demonstrated high LD of *CdX*-2 and *GATA*, resulting in the association of different haplotypes and their outcomes in the Dutch population and patients with osteoporosis [9, 22]. Accordingly, our study showed the LD between *CdX-*2 and *GATA*, illustrating a significant haplotype linked with IC.

On the other hand, our study only demonstrated high LD between GATA and *Fok*I in CH. In a prior study, *Fok*I revealed no LD with other SNP; however, the population in that study was Caucasian, while our study was Asian [22]. Therefore, the different LD findings may be a result of the differences in populations. *Fok*I is located in exon 2 of the 5’ region of the VDR gene [9]. Different *Fok*I polymorphisms cause frameshift translation, resulting in different protein synthesis [8, 23]. Previous studies revealed that *Fok*I allele and genotype were related to susceptibility to HBV infection and HCC [11–13, 24]. Therefore, the non-random selection of *Fok*I, represented in high D’ for *GATA*/FokI and various *CdX-2/GATA/Fok*I haplotypes, may lead to variability in immune control observed in patients with IC and CH phases.

According to Huang et al., *Bsm*I, *Apa*I, and *Taq*I were significantly associated with HBV patients displaying severe hepatitis flares. B/b-*Bsm*I, B/a, B/T, and B/a/T haplotypes were lower in patients with hepatitis flares than in those without [13]. By contrast, they were not at risk of HBV infection, cirrhosis, and HCC [11–13, 24–26]. In our study, none of *the Bsm*I, *Apa*I, and *Taq*I polymorphisms showed statistically significant associations with IC status.

This study was beset by several limitations. To begin with, our study is the first to describe the association between VDR polymorphisms and the IC status in chronic HBV infection. Thus, the sample size had to be estimated using the genotype frequencies cited by a previous study investigating chronic HBV infection with severe hepatitis flare. Consequently, haplotype analysis may require a larger study to validate these results. Second, a single vitamin D level was estimated using a serum sample from each participant. Some confounding factors, including nutritional status and sunlight exposure, may affect serum vitamin D levels. Lastly, our study did not evaluate other immunological parameters, such as cytokine levels and T-cell functions [24]. Thus, further studies may be needed to identify the mechanisms underlying immune control in the IC with different VDR polymorphisms.

To summarize, our results suggest that *CdX-2* G/A genotypes were independently associated with the IC status in Thai patients with chronic HBV infection. The differences between the LDs of *CdX-2/GATA* and *GATA/Fok*I in the IC and CH groups indicated that the effects of non-random selection on either group represent the variability seen in immune selection acting on the hepatitis B virus between groups.

## Supporting information

S1 Table Allele and genotype frequencies of six VDR SNPs, including *CdX-2*, *GATA*, *Fok*I, *Bsm*l, *Apa*I, and *Taq*I in healthy controls. (DOCX)

S2 Table Haplotype frequencies of six VDR SNPs, including *CdX-2*, *GATA*, *Fok*I, *Bsm*l, *Apa*I, and *Taq*I in healthy controls. (DOCX)

S3 Table Allele and genotype frequencies of six VDR SNPs including *CdX-2*, *GATA*, *Fok*I, *Bsm*l, *Apa*I and *Taq*I in patients with HBeAg positive (N 62) and negative (N 254). (DOCX)

S4 Table Haplotype frequencies of six VDR SNPs including *CdX-2*, *GATA*, *Fok*I, *Bsm*l, *Apa*I and *Taq*I in patients with HBeAg positive (N 62) and negative (N 254). (DOCX)
