# Correction: The association between vitamin D receptor polymorphism and phases of chronic hepatitis B infection in HBV carriers in Thailand

**DOI:** 10.1371/journal.pone.0303545

**Published:** 2024-05-07

**Authors:** Prooksa Ananchuensook, Sirinporn Suksawatamnauy, Panarat Thaimai, Supachaya Sriphoosanaphan, Kessarin Thanapirom, Chinachote Teerapakpinyo, Yong Poovorawan, Piyawat Komolmit

The seventh author’s name is spelled incorrectly. The correct name is: Yong Poovorawan.
