# Supplementary material for: The association between vitamin D receptor polymorphism and phases of chronic hepatitis B infection in HBV carriers in Thailand
Source: PLoS One. 2022 Dec 9;17(12):e0277907. doi: 10.1371/journal.pone.0277907 (PMC9733877; doi:10.1371/journal.pone.0277907)
Supplement: S1 Table — (DOCX) [file pone.0277907.s001.docx]

**Supplementary Table 1.** Allele and genotype frequencies of six VDR SNPs, including *CdX-2*, *GATA*, *Fok*I, *Bsm*l, *Apa*I, and *Taq*I in healthy controls

| SNPs | Allele | Frequency (%) | Genotype | Frequency (%) |
| --- | --- | --- | --- | --- |
| *CdX-2* | G  A | 59.0  41.0 | G/G  G/A  A/A | 32.2  53.6  14.2 |
| *GATA* | G  A | 2.7  97.3 | G/G  G/A  A/A | 1.1  3.3  95.6 |
| *Fok*I, | T  C | 47.5  52.5 | T/T  T/C  C/C | 22.4  50.3  27.3 |
| *Bsm*l | G  A | 91.8  8.2 | G/G  G/A | 83.6  16.4 |
| *Apa*I | T  G | 33.9  66.1 | T/T  T/G  G/G | 11.5  44.8  43.7 |
| *Taq*I | T  C | 94.3  5.7 | T/T  T/C | 88.5  11.5 |
