# Supplementary material for: The association between vitamin D receptor polymorphism and phases of chronic hepatitis B infection in HBV carriers in Thailand
Source: PLoS One. 2022 Dec 9;17(12):e0277907. doi: 10.1371/journal.pone.0277907 (PMC9733877; doi:10.1371/journal.pone.0277907)
Supplement: S2 Table — (DOCX) [file pone.0277907.s002.docx]

**Supplementary Table 2**. Haplotype frequencies of six VDR SNPs, including *CdX-2*, *GATA*, *Fok*I, *Bsm*l, *Apa*I, and *Taq*I in healthy controls

| Haplotypes | Frequency (%) |
| --- | --- |
| *CdX-2/GATA* |  |
| GG | 2.7 |
| GA | 56.3 |
| AG | 0.0 |
| AA | 42.0 |
| *GATA/FokI* |  |
| GT | 1.0 |
| GC | 1.7 |
| AT | 46.5 |
| AC | 50.8 |
| *CdX-2/GATA/FokI* |  |
| GGT | 1.0 |
| GGC | 1.7 |
| GAT | 27.0 |
| GAC | 29.3 |
| AGT  AAT | 0.0  19.5 |
| AAC | 21.5 |
| *BsmI/ApaI* |  |
| GT | 25.7 |
| GG | 66.1 |
| AT | 8.2 |
| *ApaI/TaqI* |  |
| TT | 28.1 |
| TC | 5.7 |
| GT | 66.1 |
| *BsmI/ApaI/TaqI* |  |
| GTT | 25.7 |
| GGT | 66.1 |
| ATT | 2.5 |
| ATC | 5.7 |
