# Supplementary material for: The association between vitamin D receptor polymorphism and phases of chronic hepatitis B infection in HBV carriers in Thailand
Source: PLoS One. 2022 Dec 9;17(12):e0277907. doi: 10.1371/journal.pone.0277907 (PMC9733877; doi:10.1371/journal.pone.0277907)
Supplement: S3 Table — (DOCX) [file pone.0277907.s003.docx]

**Supplementary Table 3.** Allele and genotype frequencies of six VDR SNPs including *CdX-2*, *GATA*, *Fok*I, *Bsm*l, *Apa*I and *Taq*I in patients with HBeAg positive (N 62) and negative (N 254).

| SNPs | Allele | Frequency (%) | | p-value | OR (95%CI) | Genotype | Frequency (%) | | p-value | p^HWE^ |
| --- | --- | --- | --- | --- | --- | --- | --- | --- | --- | --- |
|  |  | HBeAg (+) | HBeAg  (-) |  |  |  | HBeAg (+) | HBeAg  (-) |  |  |
| *CdX-2* | G  A | 64.5  35.5 | 56.5  43.5 | 0.105 | 1.40 (0.93-2.10) | G/G  G/A  A/A | 43.5  41.9  14.5 | 31.5  50.0  18.5 | 0.197 | 0.508 |
| *GATA* | G  A | 3.2  96.8 | 4.3  95.7 | 0.579 | 0.74 (0.25-2.18) | G/A  A/A | 6.5  93.5 | 8.7  91.3 | 0.570 | 0.792 |
| *Fok*I, | T  C | 46.8  53.2 | 45.3  54.7 | 0.764 | 1.06 (0.72-1.57) | T/T  T/C  C/C | 22.6  48.4  29.0 | 18.5  53.5  28.0 | 0.704 | 0.824 |
| *Bsm*l | G  A | 90.3  9.7 | 93.7  6.3 | 0.185 | 0.63 (0.31-1.26) | G/G  G/A | 80.6  19.4 | 87.4  12.6 | 0.168 | 0.399 |
| *Apa*I | T  G | 56.5  38.7 | 53.1  45.7 | 0.125 | 0.99 (0.63-1.57) | T/T  T/G  G/G | 56.5  38.7  4.8 | 53.1  45.7  1.2 | 0.125 | 0.663 |
| *Taq*I | T  C | 91.9  8.1 | 94.9  5.1 | 0.204 | 0.61 (0.29-1.31) | T/T  T/C | 83.9  16.1 | 89.8  10.2 | 0.190 | 0.490 |
