# Supplementary material for: The association between vitamin D receptor polymorphism and phases of chronic hepatitis B infection in HBV carriers in Thailand
Source: PLoS One. 2022 Dec 9;17(12):e0277907. doi: 10.1371/journal.pone.0277907 (PMC9733877; doi:10.1371/journal.pone.0277907)
Supplement: S4 Table — (DOCX) [file pone.0277907.s004.docx]

**Supplementary Table 4.** Haplotype frequencies of six VDR SNPs including *CdX-2*, *GATA*, *Fok*I, *Bsm*l, *Apa*I and *Taq*I in patients with HBeAg positive (N 62) and negative (N 254).

| Haplotypes | Frequency (%) | | p-value | OR | 95%CI |
| --- | --- | --- | --- | --- | --- |
|  | HBeAg (+) | HBeAg(-) |  |  |  |
| *CdX-2/GATA* | | | | | |
| GG | 3.2 | 3.9 | 0.719 | 0.82 | 0.27-2.44 |
| GA | 61.3 | 52.6 | 0.090 | 1.41 | 0.95-2.11 |
| AG | 0 | 0.4 | - | - | - |
| AA | 35.5 | 43.1 | 0.116 | 0.72 | 0.48-1.09 |
| *Corrected significant p-value is ≤ 0.0250. | | | | | |
| *GATA/FokI* | | | | | |
| GT | 0 | 1.6 | - | - | - |
| GA | 3.2 | 2.7 | 0.772 | 1.18 | 0.38-3.67 |
| AT | 46.8 | 43.6 | 0.630 | 1.10 | 0.74-1.64 |
| AC | 50.0 | 52.0 | 0.564 | 0.89 | 0.60-1.32 |
| Corrected significant p-value is ≤ 0.0250. | | | | | |
| *CdX-2/GATA/FokI* | | | | | |
| GGT | 0 | 1.8 | - | - | - |
| GGC | 3.2 | 2.0 | 0.611 | 0.73 | 0.21-2.48 |
| GAT | 31.0 | 22.5 | 0.059 | 0.65 | 0.98-2.77 |
| GAC  AGT  AGC | 30.3  0  0 | 30.2  0  0.5 | 0.325  -  - | 0.78  -  - | 0.48-1.28  -  - |
| AAT | 15.8 | 21.0 | 0.161 | 0.69 | 0.41-1.16 |
| AAC | 19.7 | 22.0 | 0.486 | 0.84 | 0.52-1.37 |
| **Corrected significant p-value is ≤ 0.0167. | | | | | |
| *BsmI/ApaI* | | | | | |
| GT | 66.1 | 69.7 | 0.443 | 0.85 | 0.56-1.29 |
| GG | 24.2 | 24.0 | 0.967 | 1.01 | 0.64-1.60 |
| AT | 9.7 | 6.. | 0.185 | 1.59 | 0.80-3.19 |
| Corrected significant p-value is ≤ 0.0250. | | | | | |
| *ApaI/TaqI* | | | | | |
| TT | 67.7 | 70.9 | 0.495 | 0.86 | 0.57-1.32 |
| TC | 8.1 | 5.1 | 0.204 | 1.63 | 0.76-3.47 |
| GT | 24.2 | 24.0 | 0.967 | 1.01 | 0.64-1.60 |
| Corrected significant p-value is ≤ 0.0250. | | | | | |
| *BsmI/ApaI/TaqI* | | | | | |
| GTT  GTC | 66.1  0 | 69.3  0.4 | 0.453  - | 0.85  - | 0.56-1.30 |
| GGT | 24.2 | 24.0 | 0.984 | 1.01 | 0.63-1.59 |
| ATT | 1.6 | 1.6 | - | - | - |
| ATC | 8.1 | 4.7 | 0.141 | 1.77 | 0.82-3.80 |
| Corrected significant p-value is ≤ 0.0167. | | | | | |
